# Genomic imprinting and assisted reproduction

**DOI:** 10.1186/1742-4755-1-6

**Published:** 2004-10-26

**Authors:** Ariane Paoloni-Giacobino, J Richard Chaillet

**Affiliations:** 1 Department of Molecular Genetics and Biochemistry, University of Pittsburgh, W1007 Biomedical Science Tower, 200 Lothrop Street, Pittsburgh, Pennsylvania 15213, USA

## Abstract

Imprinted genes exhibit a parent-of-origin specific pattern of expression. Such genes have been shown to be targets of molecular defects in particular genetic syndromes such as Beckwith-Wiedemann and Angelman syndromes. Recent reports have raised concern about the possibility that assisted reproduction techniques, such as in vitro fertilization or intracytoplasmic sperm injection, might cause genomic imprinting disorders. The number of reported cases of those disorders is still too small to draw firm conclusions and the safety of these widely used assisted reproduction techniques needs to be further evaluated.

## Introduction

The first in vitro fertilization (IVF) baby was born in 1978 and intracytoplasmic sperm injection (ICSI) was introduced in 1992 for the treatment of male infertility. Both these techniques have been continually amended and access to them improved for infertile couples. Indeed, assisted reproduction now accounts for 1% to 3% of births in developed countries [1]. Until recently, these techniques were considered accurate substitutes for natural oocyte fertilization, and were therefore regarded as safe. However, reports of children conceived by assisted reproduction techniques (ART), and presenting with congenital anomalies have been published over the last 3 years. Even though the number of reported cases indicating a link between ART and congenital anomalies is still small, the safety of these techniques needs to be evaluated. In particular, the relationship between ART and the occurrence of imprinting defects needs to be clarified.

### Epigenetics and DNA methylation

Epigenetic modifications are reversible changes of the DNA methylation pattern and chromatin structure that can affect gene expression. In many instances, epigenetic changes governing gene expression can be passed from cell to cell or from parent to offspring. Epigenetic modifications themselves might therefore explain how environmental factors modulate gene expression without affecting the genetic code. The most researched epigenetic phenomenon is DNA methylation [2].

DNA methylation is a covalent modification in which methyl groups are added to cytosine bases located 5' of guanosines (within cytosine-phospho-guanine (CpG) dinucleotides sequences). Methylation is catalyzed by the DNA cytosine-5-methyltransferase (DNA-MTase) enzyme family. Methylation induces changes in chromatin structure and is generally associated with silencing of gene expression, thus providing a way to control gene expression [3]. Indeed, methylation patterns are the result of complex interactions between de novo methylation, the maintenance of existing methylation and demethylation [4].

### Imprinting

Genomic imprinting is an epigenetic phenomenon by which the expression of a gene is determined by its parental origin. Only one allele of an imprinted gene is expressed. Imprinting is controlled by DNA methylation in such a way that a difference in methylation between the maternal and paternal alleles correlates with the different expression of the two parental alleles.

It is estimated that the total number of imprinted genes in the human and mouse genomes ranges between 100 and 200 [5]. Imprinted genes are more often grouped into clusters than scattered throughout the genome and this organization most likely reflects a coordinated way of gene regulation in a chromosomal region [6]. Two features are characteristic, although not specific, to imprinted genes. The first one is the unusual richness in CpG islands onto which imprinted patterns of methylation are placed, and the second one is the presence of clustered direct repeats near or within the CpG islands [7].

### Imprinting in development

In order to ensure that every generation receives the appropriate sex-specific imprint, the genome undergoes reprogramming. Epigenetic reprogramming has been shown to occur during gametogenesis and during preimplantation development [6]. During the development of primordial germ cells (PGC), imprinted methylation patterns are removed by a mechanism of erasure [8]. Both, passive and active demethylation may occur, although no active demethylating enzymes have yet been identified. The timing of erasure in PGCs is thought to be crucial. Studies in mice showed that erasure occurred when primordial germ cells enter into the gonads [8,9]. Erasure is followed by the establishment of sex-specific patterns of methylation during gametogenesis. Imprint establishment during gametogenesis occurs at different times in the male and female germ lines. In males it is completed by the haploid (meiotic) phase of spermatogenesis whereas in females imprint acquisition occurs in oocytes around the time of completion of the first meiotic division [5]. Furthermore, it seems that at least in oocytes, methylation might be acquired at different times (asynchronous) for different genes [5]. Epigenetic reprogramming is important for accurate development, as it controls expression of early embryonic genes, cell cleavage and cell determination in the early embryo [10].

**Figure 1:**
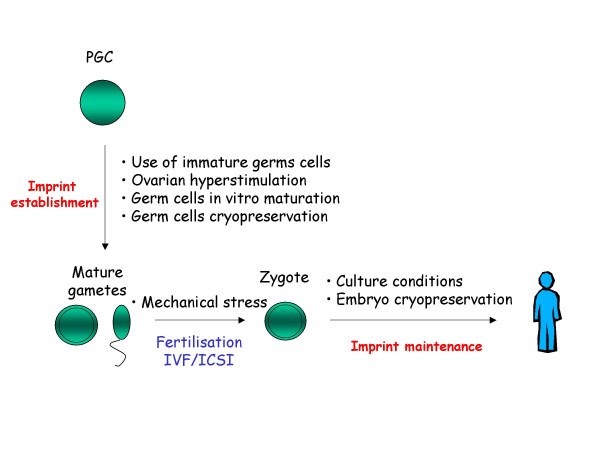
**ART and possible imprinting defects. **Possible interactions between different steps of assisted reproduction procedures and imprint establishment or maintenance through different stages of development. PGC: primordial germ cell.

Further genome reprogramming occurs during the preimplantation embryonic stage with epigenetic changes taking place through demethylation in non-imprinted genes in maternal and paternal genomes. This is followed by a genome-wide methylation at the time of implantation. The different stages of imprint establishment, maintenance and manipulations possibly disturbing them are illustrated in Figure 1. Genomic imprinting defects might indeed occur at any stage of the reprogramming process, such as during imprinting erasure, acquisition or maintenance.

The main consequence of the sex-specific establishment and maintenance of imprinted methylation patterns is the creation of maternal- and paternal-allele methylation differences (differentially methylated domains or DMDs) in or around imprinted genes. A primary DMD is established during gametogenesis and secondary DMDs develop during embryogenesis, most likely due to a direct influence of a nearby primary DMD [11].

Imprinted genes are implicated in the regulation of embryonic and fetal growth, as well as many aspects of placental function, including placental growth and the activity of transplacental transport systems [12]. Indeed, in ruminants, such as sheep and cattle, a particular overgrowth syndrome known as "large offspring syndrome" (LOS) was reported after in vitro culture of embryos. LOS is caused by abnormal methylation of the *IGF2R *gene [13]. Imprinted genes are also involved in postnatal behavior development. Based on the functions of imprinted genes, disruption of normal imprinting can have predictable consequences such as embryonic death, excessive, defective or impaired fetal growth.

### Imprinting defect syndromes in human

Several human syndromes are known to be associated with defects in gene imprinting, including Prader-Willi, Angelman, Beckwith-Wiedemann, Silver-Russell and Albright hereditary oseodystrophy syndromes [1]. Aberrant imprinting might also play a role in cancers and neuro-behavioral disorders such as autism.

The Beckwith-Wiedemann syndrome (BWS), whose frequency in the general population is about 1/14,000, is characterized by somatic overgrowth, congenital malformations and a predisposition to embryonic neoplasia. The majority of cases occur sporadically. In up to 60% of sporadic cases, the epigenetic changes occur at differentially methylated regions within 11p15.5 in a region of approximately 1 Mb. This region contains an imprinted cluster of at least 12 genes, including the paternally expressed genes *IGF2 *and *KCNQ1OT1*, and the maternally expressed genes *H19*, *CDKN1C *and *KCNQ1 *[14]. Approximately 25 to 50% of BWS patients have biallelic expression of the *IGF2 *gene, and some of these cases exhibit loss of imprinting (LOI) of *IGF2 *which is dependent on hypermethylation changes of *H19 *[14]. Approximately 50% of sporadic BWS have a loss of methylation associated to a LOI at *KCNQ1OT1*, an untranslated RNA within the *KCNQ1 *gene [15]. Some BWS cases exhibit LOI for *KCNQ1OT1 *as well as LOI for *IGF2 *[14]. It has been shown in BWS patients that aberrant methylation of *KCNQ1OT1 *is specifically associated with overgrowth and congenital defects, whereas aberrant methylation of H19 is specifically associated with an increased risk of developing tumors [16].

The Prader-Willi and Angelman syndromes (PWS/AS) are typical examples of imprinting dysregulations leading to severe neuro-behavioral disturbances. Their frequencies in the general population are approximately 1/10,000 and 1/15,000, respectively. The domain involved in these two pathologies is a 2 Mb domain on the 15q11–13 chromosomal region, including genes as *SNRPN*, *UBE3A*, *ZNF127*, *IPW *and *NDN*. The small percentage of AS cases (<5%) associated with methylation defect involves loss of methylation within the *SNRPN *imprinting center (IC) and defective expression or silencing of maternally expressed genes within this region. However, the methylation defect associated with PWS involves methylation within the *SNRPN *IC and a defective expression or silencing of paternally expressed genes within the same region. The IC comprises 2 regulatory regions: the PWS-shortest region of overlap (SRO) and the AS-SRO [17]. PWS-SRO and AS-SRO seem to operate in a stepwise way to establish imprinting during the early developmental stages [18]. Indeed, imprinting at the AS-SRO might cause maternal allele-specific repression of the PWS-SRO, preventing activation of the corresponding genes [17].

In addition, imprinting may have a wider impact on neurological development and behavior. Some reports suggest parent-specific imprinting defect in common neuro-behavioral disorders. Autism, bipolar affective disorder, schizophrenia [19] and other complex neuro-behavioral phenotypes such as alcohol abuse and audiogenic seizures [20] may be linked to imprinting disturbances. The transmission of abnormalities has been shown to be dependent upon which parent transmits the disease susceptibility. Such parent-of-origin effects on disease manifestation may be explained by a number of genetic mechanisms, one of them being genomic imprinting [21]. For instance, a lower age of onset of symptoms following paternal inheritance of one subtype of schizophrenia and following maternal inheritance of Tourette's syndrome suggests that imprinted genes are involved in the pathophysiology of these syndromes. Similarly, parent-specific components for late-onset Alzheimer's disease (paternal-specific component) or familial neural tube defects (maternal-specific component) have been described [20].

### Cases of defective imprinting in ART conceptions

Prior to the establishment of sex-specific imprints in male and female germ cell lineages, imprints are erased. After erasure of the pre-existing imprints, the timing of acquisition of imprints is significantly different between the two germ lines [6]. In the female germ line, methylation occurs in the postnatal growth phase while oocytes are arrested at the diplotene stage of prophase I [22], whereas during spermatogenesis, methylation takes place before meiosis [23]. Maternal imprints are continually established as oocytes mature in females, and paternal imprints are established as long as spermatogonia proliferate in males. Thus, paternal imprints seem to be established earlier than maternal ones. It has been shown that this sex-specific methylation is intrinsic and cell-autonomous, and is not due to any influence of the genital ridge somatic cells, or gonadal environment on the primordial germ cells [24]. Imprinting defects in the course of assisted reproduction could theoretically occur during several stages of the methylation erasure/re-methylation process in male and female germ cells as well as during the early stages of in vitro embryonic development.

The first baby conceived by IVF was born 26 years ago. Intracytoplasmic sperm injection (ICSI), developed approximately 10 years ago, was seen to be the reproductive solution for severe male infertility. Several studies have established the general safety of both IVF and ICSI [25]. Nevertheless, it was recently reported that IVF and ICSI may be associated with an increased risk of major birth defects. Schieve et al. [26] studied 42 463 infants conceived with assisted reproductive techniques and reported a higher occurrence of low (less-than-or-equal 2500 g) and very low (less-than 1500 g) birth weight in this group compared to the control population of children naturally conceived. Hansen et al. [27] in a study on 837 infants conceived by IVF and 301 infants conceived by ICSI, reported rates of major birth defects (musculoskeletal, cardiovascular, urogenital, gastrointestinal, central nervous system, metabolic and poorly defined ones), as high as 9.0% for IVF and 8.6% for ICSI conceptions, compared to 4.2% reported for natural conceptions. A possible link with imprinting disturbances was not considered by the authors. These results were in part due to the increase in multiple pregnancies, known to be associated with ART, but also due to a higher rate of low birth weight babies among singleton pregnancies. In addition to these associated defects, a higher incidence of sex-chromosome aneuploidy has also been reported in ART conceptions [27].

DeBaun et al. [28] recently reported 7 cases of BWS conceived by ART, 6 of those showing an imprinting defect at *KCNQ1OT1 *or H19. By comparing this rate of ART-conceived BWS to the rate of ART in the general population during the same time period, sporadic cases of BWS were approximately six times more likely to have been conceived by ART than by natural conception. The authors suggested that causative factors may include the in-vitro culture conditions or the exposure of the gametes or embryos to specific media or growth factors.

Maher et al. reviewed a different set of sporadic BWS cases and looked for an association with ART [29]. Six out of the 149 BWS cases examined were conceived by ART, and 2 of these had a *KCNQ1OT1 *loss of imprinting as the causative molecular defect. Indeed, when compared to the incidence in the general population, ART had a four-fold greater likelihood of being associated with BWS than natural conception. The cases reported by DeBaun et al. [28] and Maher et al. [29] were recruited through registries of BWS patients. However, parents with BWS babies born after ART may be more likely to join BWS registries, which could introduce bias when using these registries.

Recently, a case-control study analyzed the frequency of BWS in 1'316'500 live births and 14'894 babies born after an IVF procedure [30]. The risk of BWS was reported to be 9 times higher in the IVF population compared to the general population.

Cox et al. [32] and Orstavik et al. [33] reported a total of 3 children with Angelman syndrome conceived by ICSI. In all 3 cases, AS was due to loss of imprinting within *SNRPN *gene at 15q11–13. Considering that the occurrence of AS in the general population is about 1/15,000 and that <5% of cases are due to epigenetic imprinting defects, these reports suggest that the predominant abnormalities seen in ART are epigenetic rather than genetic.

However, no evidence of abnormal methylation patterns at 15q11–13, the locus linked to the pathogenesis of AS and PWS, was found in 92 children conceived by ICSI [31].

### Why might ART be harmful for the imprints

For assisted reproduction by intracytoplasmic sperm injection (ICSI), the injection of a spermatozoon into the ovum by micro-manipulation bypasses several of the steps involved in fertilization. However, in male germ cells, it seems that the paternal imprints are well established in the mature, meiotic stages of spermatogenesis. Furthermore, round spermatid microinjections have confirmed that paternal imprints are completely established in primary spermatocytes [34]. This point is relevant to the recent use of ICSI using round spermatids. Manning et al. [35] have analyzed the methylation pattern in immature testicular sperm cells at different developmental stages at the 15q11–13 imprinted region and reported that the ejaculated spermatozoa and elongated spermatids had completed the establishment of paternal methylation imprints. However, spermatozoa used for ICSI generally originate from men with abnormal semen parameters that may have had adversely affected the establishment of imprints. Moreover, immature spermatozoa for ICSI can also be directly collected from the testes of infertile males. It has been hypothesized that spermatozoa from men with fertility problems contain a higher number of gametes with chromosomal abnormalities [36]. A defect in gene imprinting can be considered as a possible sperm abnormality. Indeed, a recent report has analyzed the imprinting of two opposite imprinted genes (*MEST *and *H19*) in spermatozoon DNA from normozoospermic and oligozoospermic patients. The data presented suggest an association between abnormal genomic imprinting and hypospermatogenesis [37]. Theoretically, it is possible that freezing of mature sperm or the cryoprotectants used might disturb the established male imprints in mature spermatozoa or round spermatids.

Women with a variety of fertility problems, such as ovarian failure and/or hormonal disturbances, may be more prone to produce gametes with inherent imprinting defects because of the establishment of maternal imprints during the final phase of oocyte growth and meiotic maturation.

Although biologically plausible, this is purely speculative at the moment.

In addition to the theoretical possibility that there may be innate defects in oocytes used in ART, the in vitro treatment of oocytes and embryos during ART procedures might affect the establishment of imprints in female germ cells. For example, superovulation or in vitro maturation of oocytes might affect the establishment of the complete array of normal maternal imprints. Oocytes used for assisted reproduction usually originate from women who undergo hormonal hyperstimulation protocol followed by fertilization in vitro. It is not clear to date if the clinical use of high doses of gonadotrophins might alter imprint acquisition. Gonadotrophins might cause the premature release of immature oocytes that have not completed the establishment of their imprints, and establishment may not be completed during in vitro maturation. Shi and Haaf [38] determined the possible incidence of abnormal methylation patterns in mice embryos from superovulated compared to non-superovulated female mice. An immunostaining method was used to assess the overall extent of genomic cytosine methylation and reported abnormal methylation patterns in 2-cell embryos from superovulated females as compared to non-superovulated ones. Kerjean et al [39] explored in mice whether maternal imprinting progresses normally when oocytes are cultured in vitro. The authors analyzed the DMDs of 3 imprinted genes and reported that indeed *in vitro *culture affected imprint establishment and might lead to loss of methylation at certain imprinted loci, such as *IGF2R *and gain of methylation at other loci, such as *H19*. However, to our knowledge, no data concerning the possible effects of ovarian hyperstimulation on imprinting in humans is available yet.

Potential disruption of normal imprinting could result from the in vitro manipulation of early stage embryos. In vitro culture with the use of slightly different culture media led to decreased fetal viability and imprinting disturbances in mice. Doherty et al. [40] first reported the differential affects of culture media in preimplantation mouse embryos at the *H19 *imprinted gene. The loss of methylation at *H19 *gene was associated with culture in Whitten's media, resulting in LOI in the imprinting control domain upstream of the start of *H19 *transcription. Khosla et al. [41] examined mouse preimplantation mouse embryos cultured in different culture media and transferred into recipient mothers. Fetal development as well as the expression pattern of imprinted genes, including the *IGF2 *and *H19 *genes, was influenced by the addition of fetal calf serum (FCS) in the culture media. The mechanism by which culture media and other gamete or embryo handling might induce defects and lack of maintenance of methylation at imprinted loci is not clear. It may be due to the facilitation of removal of methyl groups on cytosine bases or the disturbance of the gamete development leading to incompleteness of imprint erasure and/or establishment [10]. Furthermore, cryopreservation of embryos could potentially affect the cytoskeleton, chromatin structure and the availability of methylating and/or demethylating enzymes during preimplantation development. However, it is not known at present if culture of human preimplantation embryos in different media or over longer periods – might lead to disturbances in genomic imprinting.

Disturbances in imprinting could affect the germline cells of the embryo conceived by assisted reproduction and the problems of imprinting might occur in the offspring of the subsequent generation [10]. Follow-up of these individuals may give important information about the possible risks associated with ART.

### Imprinting and placenta

A critical way of regulating intrauterine development is through placental function and growth. Most imprinted genes are expressed in fetal and placental tissues, and are involved in fetal growth [12]. In general, paternally expressed imprinted genes enhance fetal growth whereas maternally expressed imprinted ones suppress it [6]. Among the genes expressed in the placenta, the *MASH2 *gene was shown to regulate the development of spongiotrophoblast [42]. Igf2 transcripts are found specifically in the labyrinthine trophoblast [43], and *ASCL2 *is a transcription factor expressed in the spongiotrophoblast and labyrinthine layers [5]. Indeed, mice with deletions of *IGF2 *and *ASCL2 *genes showed fetal growth restriction and death during embryonic development [43,42].

In humans, several imprinting disorders are associated with intrauterine growth restriction (IUGR) [44]. Studies on human placental imprinted genes and on the different roles of the maternally and paternally expressed genes are certainly needed to understand the placenta's role in normal embryonic and fetal development. Furthermore, analyses of placental samples obtained after ART conceptions might provide answers to some important questions about the possible links between ART and genomic imprinting.

## Conclusion

Concern has been raised about the possible increased incidence of genetic syndromes due to imprinting defects in children conceived by assisted reproduction. In particular, experimental reports in mice have raised the question that some of the steps involved in these techniques, such as ovarian hyperstimulation or certain culture media for in vitro culture of embryos might be detrimental to the formation of genomic imprints. In order to be able to adequately counsel infertile couples enquiring about ART, solid evidence from large, well-designed studies as well as cautious long-term evaluation of the safety of these techniques need to be available. Although the unraveling of the mechanisms underlying genomic imprinting is only at the beginning, there is a clear need to investigate and better understand the regulation of this process during fecundation and embryogenesis.

## Competing interests

The authors declare that they have no competing interests.

## Authors' contributions

Both authors contributed to the writing of this review and both read and approved the final manuscript.
